# The Use of a Thermal Process to Produce Black Garlic: Differences in the Physicochemical and Sensory Characteristics Using Seven Varieties of Fresh Garlic

**DOI:** 10.3390/foods10112703

**Published:** 2021-11-05

**Authors:** Jan Bedrníček, Ivana Laknerová, František Lorenc, Priscila Probio de Moraes, Markéta Jarošová, Eva Samková, Jan Tříska, Naděžda Vrchotová, Jaromír Kadlec, Pavel Smetana

**Affiliations:** 1 Department of Food Biotechnologies and Agricultural Products’ Quality, Faculty of Agriculture, University of South Bohemia in České Budějovice, Studentská 1668, 370 05 České Budějovice, Czech Republic; bedrnj00@zf.jcu.cz (J.B.); lorenf00@zf.jcu.cz (F.L.); kadlec@zf.jcu.cz (J.K.); smetana@zf.jcu.cz (P.S.); 2 Food Research Institute Prague, Radiová 1285/7, 102 00 Praha 10—Hostivař, Czech Republic; ivana.laknerova@vupp.cz; 3 Department of Food Science, School of Food Engineering, University of Campinas, Monteiro Lobato No. 80, Campinas 13083-862, Sāo Paulo, Brazil; priscilaprobiomoraes@gmail.com; 4 Department of Plant Production, Faculty of Agriculture, University of South Bohemia in České Budějovice, Na Sádkách 1780, 370 05 České Budějovice, Czech Republic; jarosm08@zf.jcu.cz; 5 Global Change Research Institute CAS, 603 00 Brno, Czech Republic; triska.j@czechglobe.cz (J.T.); vrchotova.n@czechglobe.cz (N.V.)

**Keywords:** garlic, ageing process, antioxidant activity, bioactive compounds, organoleptic properties, HPLC-MS/MS analysis

## Abstract

Black garlic (BG) is a product originating from fresh garlic (FG) and substantially differs in many aspects from FG due to the process called ageing. During this thermal process, the health-promoting properties of FG are enhanced, and the sensory traits are altered. However, very little is known about how the physicochemical properties of different FG varieties affect these properties of BG. Thus, the aim of this study was to investigate the influence of seven FG varieties subjected to the thermal process on the physicochemical parameters of BG. To prepare the BG samples, a fifteen-day ageing process involving a temperature gradient ranging from 30 to 82 °C was used. It was found that the antioxidant activity, the total polyphenol content, and the total soluble solids increased during ageing, while the pH level, moisture content, and lightness decreased in all the garlic varieties. The varieties of garlic differed in the studied traits significantly, both before (FG) and after ageing (BG). In the sensory analysis, significant differences between the BG varieties were observed only in the pleasantness of texture, while the remaining sensory descriptors (pleasantness of color, odor, taste and intensity of the garlic aroma, and overall acceptability) were not affected by variety. The correlations suggest that most of the FG’s studied parameters in this study do not correlate with the properties of BG and cannot be used for the prediction of the quality of BG. Additionally, HPLC-MS/MS analysis revealed substantial changes in the composition of low molecular compounds.

## 1. Introduction

Black garlic (BG) is a functional food originating from Asia, specifically from China, South Korea, and Japan. Although BG is botanically *Allium sativa* L., it differs from fresh garlic (FG) in many aspects. It is processed using higher temperatures, ranging between 60–90 °C, with approximately 80% relative air humidity for a long period of time (10–90 days) without using any other ingredients or additives. This thermal process is called ageing and during this process the fresh white garlic gains many new properties, such as enhanced biological activity, especially antioxidant activity, and altered sensory properties because the FG’s color turns from white to black, the texture changes to very soft or jelly-like, and the taste is very mild with sweet and sour tones [1]. Moreover, antimicrobial activity was investigated by Botas et al. [2] who found that BG had stronger antimicrobial activity than FG.

As reported previously, BG with excellent sensory and antioxidant properties is obtained using a temperature around 70 °C and 80% relative air humidity. Lower temperatures do not lead to the black color, and a pungent aroma is still present. A higher temperature than 85 °C accelerates the process but could give a bitter aroma to the BG, suggesting the presence of undesirable compounds [3].

BG is popular in Asia [4] and is now attracting more attention in Europe, including the Czech Republic. It is becoming an ingredient in luxury cuisine and part of a healthy diet [5].

BG has been intensively studied in the last two decades. The research has been, nevertheless, mostly focused on the physicochemical properties of BG [6]; its in vivo and in vitro effects; its bioavailability [7,8]; the influence of the processing conditions (temperature, time, and relative air humidity); and the quality of the BG [3], including pretreatment steps, such as freezing [9] and the use of hot steam [10].

Many factors influence the quality parameters of FG, such as origin, growing location, season, climate, and cultivation practice, as well as the variety, which plays a pivotal role [11]. Moreover, the different FG qualities could lead to different qualities of BG. Czech FG is assessed positively by experts and consumers due to its strong garlic aroma and taste [12]. In addition, the authors reported that the pungency of Czech varieties was higher than in European samples and much higher than in non-European. A typical, strong, pungent aroma should not be present in BG. Thus, it remains unclear whether the pungent varieties (e.g., those originating from the Czech Republic) are suitable for the preparation of BG.

Very little is known about the influence of garlic variety on BG quality. Up to now, only two papers have been published studying the influence of garlic variety on the properties of BG, with only one focusing on the quality of BG originating from conventional and organic cultivation [13,14,15]. However, these papers studied only three, six, and two varieties, respectively.

The aim of this study was to describe the influence of seven Czech FG varieties and the thermal ageing technology on the physicochemical properties and sensory qualities of the BG prepared from them. Moreover, the study targeted identification of the main biologically active compounds before and after ageing.

The results could be helpful for producers of BG to select garlic that has the highest quality and can also contribute to the knowledge of how the parameters affect the general quality of the BG.

## 2. Materials and Methods

### 2.1. Chemicals

All chemicals were purchased from Sigma-Aldrich, Prague, Czech Republic: acetic acid (purity ≥ 99%); sodium acetate (purity ≥ 99%); acetonitrile (LC/MS grade); methanol (LC/MS grade); formic acid (LC/MS grade, 98–100%); ferric chloride; hydrochloric acid (37%; HCl); sodium carbonate; 2,4,6-tris(2-pyridyl)-1,3,5-triazine (TPTZ, purity ≥ 98%); 2,2-diphenyl-1-picrylhydrazyl (DPPH); Trolox (purity ≥ 97%); and Folin–Ciocalteau’s phenol reagent and gallic acid (97.9%).

### 2.2. Plant Material

In this study, seven varieties of Czech garlic (listed in the Czech State Variety Book) were used: Bjetin, Vekan, Lan, Havel, Rusák, Havran, and Lukan. All of them were purchased from a local garlic grower (Mihulková-Hradecký “Český česnek z Podkrkonoší”, Czech Republic). The varieties were of the winter and bolting type (except for the Lukan, which was non-bolting) and were planted in the autumn (in the middle of October) of 2018 and harvested at the turn of the month of June and July 2019 in the Semily district (ca. 360 m above the sea level) in the Czech Republic. The average annual temperature in this area is around 7 °C with precipitations of 700 mm. The garlic samples were transported to the laboratory fresh and disease-free after harvesting. These FG samples were used for the manufacturing of BG or directly for physicochemical analyses: determination of the moisture content, soluble solids, pH level, total polyphenolic content, antioxidant activity, and texture and color analysis. Furthermore, the variety Rusák was used to determine the main biologically active compounds by means of high-performance liquid chromatography and tandem mass spectrometry (HPLC-MS/MS).

For each chemical analysis, five randomly selected bulbs of each variety were taken, and two cloves from each bulb were peeled (ten cloves per variety in total); these are assigned as “peeled garlic cloves” later in the text.

### 2.3. Black Garlic Processing

The BG was made from FG according to the patented method described by Vácha et al. [16]. Briefly, approximately 550 g of FG of each of the above-mentioned varieties was placed into a polyamide/polyethylene vacuum bag. Then, the samples were vacuum-packed using ETA Freshie 1762 (ETA a.s., Prague, Czech Republic). The purpose of using vacuum plastic bags is that the BG can be produced in an oven or a dryer without any humidity regulation because the high relative humidity needed for the ageing process is substituted by the water contained in the FG. Thus, the bags prevent or minimize moisture evaporation from the garlic bulbs.

The bags with the FG samples were placed into a laboratory oven (Memmert UFE 400, Memmert GmbH + Co. KG, Schwabach, Germany) to begin the process of ageing at the same time. The only parameter that was controlled was the temperature, as follows: the first stage took three days, and the temperature was set at 82 °C; in the second stage, the temperature was decreased to 30 °C for one day; and finally, the temperature was increased to 72 °C in the last stage for 11 days. Thus, the whole ageing process took 15 days. At the beginning of the ageing process, the highest temperature was used to rapidly accelerate ageing and inactivate some enzymes, such as alliinase (EC 4.4.1.4.). Due to the excessive water evaporation from the garlic bulbs, a low temperature was set in the second stage of the process. It caused the water to be condensed back to the surface of the bulbs. Afterwards, a temperature of around 70 °C in the third stage was set because it was optimal for obtaining BG with excellent properties [3].

Then, the samples from each variety were collected by the same sampling procedure as described for the FG samples. Afterwards, the black, aged samples were used for the same analyses as in the case of the fresh samples. The BG samples were additionally used for sensory analysis.

### 2.4. Chemical Analyses

#### 2.4.1. Moisture-Content Analysis

The peeled garlic cloves were finely homogenized in a kitchen mixer, and 5 g of the sample was measured gravimetrically using a moisture analyzer (Kern RH 120-3, Fisher Scientific s.r.o., Pardubice, Czech Republic). The measurement was repeated three times (*n* = 3).

#### 2.4.2. pH Measurement

The peeled garlic cloves were homogenized using a kitchen blender, and the pH of garlic samples was measured using a pH meter equipped with a spear-type electrode (HC 124, Fisher Scientific, Pardubice, Czech Republic). An analysis of each group was conducted three times (*n* = 3).

#### 2.4.3. Determination of Total Soluble Solids

Approximately 10 g of the peeled garlic cloves was blended with 90 mL of deionized water in a glass tube using a laboratory homogenizer (Ultra-Turrax T25, IKA-Werke GmbH & Co. Kg, Staufen, Germany). The mixture was then analyzed for total soluble solids using an optical refractometer (ATC 0–32%, Fisher Scientific s.r.o, Pardubice, Czech Republic), previously calibrated with deionized water. °Brix were then calculated using a dilution factor. An analysis for each group was conducted three times (*n* = 3).

#### 2.4.4. Determination of Antioxidant Activity

To determine the antioxidant activity of the FG and BG samples, two spectrophotometric methods were selected: DPPH (2,2-diphenyl-1-picrylhydrazyl), to evaluate the free radical scavenging ability and FRAP (ferric reducing antioxidant power), to assess the reducing ability. The first step was the extraction of a sample. The peeled garlic cloves were homogenized, and 0.2 g was placed in a 15 mL centrifuge tube, then 9.8 mL of 90% methanol (*v*/*v*) was added to the sample. This mixture was shaken in a laboratory shaker for 10 min, and then it was centrifuged at 7000 rpm for 15 min at 5 °C. The supernatant was collected and immediately used for the determination of antioxidant activity. Two extractions per group were conducted, and each extract was measured twice (*n* = 4).

The DPPH method was conducted according to a slightly modified method described by Brand-Williams et al. [17]. The extracted sample (100 µL) was added to 4 mL of methanolic solution of DPPH (27.5 µg/mL). Afterwards, this mixture was kept for two hours in the dark at room temperature. Then, the absorbance at 515 nm was measured against a blank (DPPH solution without sample). Trolox was used to prepare a calibration curve. The results are expressed as Trolox equivalent (TE) per gram of sample on a dry matter (DM) basis (g TE/kg DM).

A slightly modified FRAP method was assessed according to Dudonné et al. [18]. Firstly, the FRAP reagent was prepared as follows: 100 mL of 300 mM (pH 3.6) acetate buffer was mixed with 10 mL of 10 nM TPTZ in 40 mM HCl and with 10 mL of ferric chloride (10 mM). After this, 0.1 mL of sample extract was pipetted to 4 mL of FRAP reagent. The reaction mixture was kept in the dark for 30 min at 37 °C, and then the absorbance was measured against a blank (acetate buffer) at 593 nm. Trolox was used as a standard; thus, the results are expressed as g TE/kg DM.

#### 2.4.5. Determination of Total Polyphenol Content

Firstly, polyphenols from the garlic samples were extracted. The peeled garlic cloves (fresh and black) were homogenized, and 5 g was weighed into a glass tube. Then, 50 mL of 50% methanol was added. Additionally, the mixture was thoroughly homogenized using a laboratory mixer (Ultra-Turrax T25, IKA-Werke GmbH & Co. Kg, Staufen, Germany) for 30 s. Then, the tube was put into an ultrasound bath and was extracted for 15 min. Afterwards, an aliquot was pipetted into a centrifuge tube and was centrifuged at 4000 rpm for 15 min. The supernatant was stored at −18 °C until analysis. One extraction per group was conducted, and the extract was analyzed three times (*n* = 3).

The total polyphenolic content was spectrophotometrically determined according to the method of Lachman et al. [19]: 35 µL of the sample extract was pipetted into a disposable 4.5 mL plastic cuvette; then, 175 µL of Folin–Ciocâlteu reagent, 3465 µL of water, and 525 µL of 20% sodium carbonate were added. This reaction mixture was then incubated at room temperature for two hours in the dark. The absorbance was read at 765 nm, and gallic acid was used as a standard for the preparation of a calibration curve. The results are expressed as mg of gallic acid equivalent (GAE) per kg of sample DM (g GAE/kg DM).

#### 2.4.6. HPLC-MS/MS Non-Targeted Analysis of Fresh and Black Garlic

Extraction was conducted according to Zhang et al. [20], with modifications. The peeled garlic cloves were homogenized in a kitchen mixer, and 5 g of the sample was immediately put into a glass tube; then, 50 mL of 100% methanol was added. The sample with methanol was additionally homogenized using a laboratory mixer (Ultra-Turrax T25, IKA-Werke GmbH & Co. KG, Staufen, Germany) for 30 s. Afterwards, the mixture was extracted in an ultrasound bath for 15 min. Then, an aliquot was transferred into a centrifuge tube and centrifuged at 4000 rpm for 15 min. The supernatant was collected and stored at −18 °C until analysis. Five µL of properly diluted sample was injected into the HPLC system (Dionex UltiMate 3000; Dionex, Sunnyvale, CA, USA) coupled with a triple quadrupole mass spectrometer (Agilent 6420; Agilent Technologies Inc., Santa Clara, CA, USA). The chromatographic separation was achieved by the Phenomenex Kinetex column (C18, 2.6 µm, 150 × 2.1 mm) maintained at 35 °C. The mobile phase A consisted of 5% acetonitrile with 0.5% formic acid, and B consisted of 100% acetonitrile. The flow rate was set at 0.2 mL/min and the gradient involved a linear increase in the mobile phase B from 5 to 80% within 30 min. After this, solvent B decreased to 5% within 10 min to be prepared for the next injection. Identification of the separated compounds was assessed using a mass spectrometer equipped with an electrospray ionization source (ESI) operating in positive ionization mode with the following settings: the drying gas (N_2_) temperature of 300 °C was at a flow rate of 11 L/min, the nebulizing gas pressure was 35 psi, and the capillary voltage was set to +4kV. The fragmentor, cell accelerator, and collision energy were 135, 7, and 20 volts, respectively.

### 2.5. Physical Properties Analysis

#### 2.5.1. Color Analysis

The surface color of the FG and BG peeled cloves was analyzed in the CIE L*a*b* system using a spectrophotometer ColorEye XTH (GretagMacbeth, New Windsor, NY, USA). For each sample, three independently chosen cloves were selected and analyzed (*n* = 3).

#### 2.5.2. Texture Analysis

The hardness measurement of the FG and BG cloves was accomplished by using texture analyzer TA.XT Plus (Stable MicroSystems, Godalming, UK). The analyzer was equipped with a knife probe that cut the garlic cloves, and the maximal force needed to cut the cloves into halves was recorded. The following settings of the texture analyzer were applied: pre-test speed: 3 mm/s; test speed: 2 mm/s; and post-test speed: 10 mm/s. Four cloves from each group (*n* = 4) were randomly selected, and the results are expressed as newton (N).

### 2.6. Sensory Analysis of Black Garlic Samples

The sensory profile of the BG varieties was tested by sensory analysis. Seven panelists, who had been well trained in the principles and the concept of sensory evaluation, were involved in this experiment. One day before the sensory analysis, the panelists received commercially available BG cloves to get familiar with their sensory attributes.

At sensory analysis, approximately 5 g of BG per variety, corresponding to at least three cloves, were put into a 50 mL plastic container. The containers, randomly marked with a number, were served on a plate to each panelist to compare them simultaneously. The hedonic evaluation of taste, color, texture, overall acceptability, and intensity of the garlic aroma were recorded on a 10 cm unstructured hedonic scale (0 = dislike extremely, 10 = like extremely for taste, smell, color, and overall acceptability; 10 = very intense, 0 = not present for the intensity of the garlic aroma) by the same panel. The panelists then placed a mark on the scale to indicate the pleasantness or intensity of each characteristic. The values for all descriptors of BG obtained from each panelist were averaged (*n* = 7). Water was served as a taste neutralizer and was drunk between the judging of the following sample.

### 2.7. Statistical Analysis

The results of all the analyses are presented as mean ± standard deviation. Two-way ANOVA (analysis of variance) was used to determine statistically significant differences between varieties (factor one) and between the FG and BG samples (ageing process—factor two) in all the physicochemical analyses. To evaluate the statistically significant differences between the BG varieties in the sensory analysis, one-way ANOVA was used. After both ANOVAs, Tukey’s HSD post hoc test was assessed for group comparisons. To estimate the associations between garlic sample properties (soluble solids, antioxidant activity, pH, total polyphenol content, etc.), Pearson’s correlation coefficient was used. Statistical evaluation was performed in Statistica CZ, version 12 (StatSoft CR) and the differences were considered significant if *p* < 0.05.

## 3. Results and Discussion

### 3.1. The Effects of the Ageing Process and Variety on Moisture, pH, and Total Soluble Solid Content of Fresh and Aged Garlic Samples

The ageing process substantially affects the chemical properties of garlic [1]. The results of this study also prove the significant changes in the chemical parameters. The moisture, pH, and total soluble solid content of all the FG and BG varieties are presented in Table 1.

The moisture content of the FG varieties ranged between 57.04 and 65.34%, with an average value of 59.57% and between 33.52 and 45.55%, with an average value of 38.26% for the garlic samples after ageing (BG). Thus, the differences between varieties (both before and after ageing) were statistically significant (*p* < 0.05). These values are typical for FG originating from the Czech Republic, where the moisture content is usually between 55 and 70% [12]. When compared with garlic from other countries, samples originating from South Korea have an average moisture content of 63.43%, whereas Chinese samples have 66.76% [21]. Moreover, Shin et al. [22] reported that Chinese garlic samples had a moisture content higher than 70% on average. This indicates that garlic from East Asia has a higher moisture content. Regarding BG moisture, Sunanta et al. [14] reported that BG had an average moisture content of 30%. However, the authors also reported that garlic originating from China had moisture of 53.12% after ageing, which is much higher than our observation.

The ageing process resulted in an approximately 37% loss of water (from 59.57 to 38.26%), and the decrease was statistically significant (*p* < 0.05). The evaporation of water during the processing of BG is a common phenomenon. However, as stated by Xiong et al. [23], water evaporation during ageing is strongly dependent on the processing conditions, mainly the relative air humidity as well as the temperature.

The ageing process caused a significant (*p* < 0.05) decrease of pH in all the garlic samples, from approximately 6.04 to 4.39. Other authors [6,15] have reported a much lower pH, reaching values of 3.3. However, they processed garlic samples for more than 30 days. A low pH resulting from acidic conditions in BG can be caused by the formation of acids, such as formic, acetic, pyroglutamic, 3-hydroxypropionic, and succinic, which were not detected in FG, as reported by Liang et al. [24]. Although there were statistical differences (*p* < 0.05) between the varieties in pH, the range of values was relatively small in the FG and BG samples. The pH ranged between 5.99 and 6.11 for the FG samples and 4.24 and 4.48 for the BG samples. Our results are in line with Toledano Medina et al. [15], who also reported that the pH of garlic is affected by variety.

Another observed parameter in the garlic samples was total soluble solid content. The values in the FG samples varied between 30.00 to 39.67 °Brix (the average value was 36.38 °Brix). Much higher values were measured in the garlic samples after ageing, with an average value of 53.67 °Brix (ranging between 50.00 and 60.00). Thus, the ageing process had a significant (*p* < 0.05) effect on this parameter. Moreover, the variety significantly (*p* < 0.05) influenced the total soluble solid content. These results are in accordance with other authors [13,25]; however, they reported lower values, both in the FG and the BG samples. The increase in soluble solids is probably caused by the hydrolysis of polymeric compounds at a higher temperature into simpler compounds, e.g., proteins into peptides or amino acids and especially polysaccharides into oligosaccharides with a lower degree of polymerization and di- or monosaccharides [1,26].

### 3.2. The Effects of the Ageing Process and Variety on Antioxidant Activity and Total Polyphenol Content of Fresh and Aged Garlic Samples

Garlic possesses antioxidant activity due to the presence of many substances, such as polyphenols and organosulfur compounds, but the activity is strongly enhanced after the ageing process of garlic [27]. The antioxidant activity of the FG and BG samples is presented in Table 2. 

The BG samples exhibited much higher antioxidant activity, both free radical scavenging activity (DPPH assay) and reducing properties (FRAP assay), compared to the FG samples. Thus, the ageing process is a significant (*p* < 0.05) factor influencing antioxidant activity. There was an almost seven-fold increase in the DPPH assay, from 1.9 (average of FG) to 12.93 g TE/kg DM (average of BG). A very similar trend was observed for the FRAP assay, but the increase was even more significant, from 0.5 to 12.89 g TE/kg DM. The DPPH was also found to be variety dependent as significant (*p* < 0.05) differences were found between varieties (in the FG and also the BG samples). Nevertheless, no statistical differences were found between the FG samples in terms of the FRAP assay, but the BG samples differed significantly.

It was reported that some of the compounds possessing antioxidant activity in garlic might be polyphenols that can be determined by the spectrophotometric method using Folin–Ciocâlteu phenol reagent [28]. Although its selectivity and specificity are questionable [29,30], it is one of the most frequently used assays for the determination of such compounds, not only in FG and BG [6,28,31]. A relatively low concentration of total polyphenols (3.82 g GAE/kg DM on average) was found in the FG samples, as can be seen in Table 2. It should be noted that according to Pilluza and Bullitta [28] garlic had the lowest concentration of total polyphenols among 24 examined plants.

It was an interesting finding that there were no statistically significant differences (*p* > 0.05) between the FG varieties, although some distinctions were noted. The presented results of this study show that there was approximately a 3.6-fold increase in the total polyphenol content after ageing (14.03 g GAE/kg DM on average), as was also found in the previous literature [4]. On the other hand, statistically significant differences (*p* < 0.05) were found between the varieties after ageing. Although the result is in accordance with an earlier report, it remains a question what compounds in BG react with the Folin–Ciocâlteu phenol reagent. Thus, in our opinion, this phenomenon should be taken into account in further research.

Total polyphenol content often positively correlates with antioxidant activity [28], but the association between total polyphenols and antioxidant activity (DPPH and FRAP assays) in samples after ageing (BG) was found to be very small (r = 0.408 for DPPH and 0.256 for FRAP, respectively, *p* > 0.05). This also suggests that compounds other than polyphenols can contribute to the antioxidant activity, e.g., organosulfur compounds or products of the Maillard reaction. It should also be mentioned that the total polyphenol content could be affected by the particular temperature gradient. Xiong et al. [23] reported that if garlic is aged at 90 °C, the total polyphenol content rises faster than if the temperature is set at 70 °C. According to the author, if a higher temperature was set, then after five days, the polyphenol content decreased. Thus, it is advisable to process BG at 70 °C for at least 15 days.

### 3.3. HPLC-MS/MS Non-Targeted Analysis of Low Molecular Weight Compounds in Fresh and Black Garlic Samples

In addition to spectrophotometric analyses (TPC, DPPH, and FRAP), we decided to identify some of the low molecular weight compounds in FG and BG using HPLC-MS/MS apparatus to establish differences in the samples before and after ageing. The Rusák variety was chosen for this purpose. Compounds with high intensity (response) or significant changes due to the ageing process were subsequently identified. The identification of the compounds was based on the monitoring of molecular mass, production of MS/MS fragments, retention times, and comparison with the data from available literature [32,33,34,35,36,37] and with the help of online mass spectra databases [38,39].

Data were obtained only in positive ionization mode as bad results were achieved with negative ionization. The relative concentration change of the identified compounds on a dry matter basis (peak area vs. dry matter) was also calculated. It was expressed only as “increase” or “decrease” due to the uncertainty of the detector linearity; thus, fold change is not presented. The results of the MS/MS analysis showing tentatively identified compounds in the Rusák variety before (FG) and after ageing (BG) are presented in Table 3.

Twelve compounds in total were identified in the methanolic extracts, including amino acid, amino acid derivative, fructooligosaccharides, organosulfur compounds, and derivatives belonging to β-carbolines.

The compound with the highest *m*/*z* (1515 [M + K]^+^) was identified as fructooligosaccharide residue with a degree of polymerization (DP) 9 as it showed a typical fragmentation pattern (*m*/*z* of 1353, 1191, 1029, 867, 705, 543, 381) with a mass shift of 162 Da corresponding to the loss of fructose moiety. Other fructooligosaccharides identified in the extracts were fructofuranosyl nystose (*m*/*z* 867, DP = 5), nystose (*m*/*z* 705, DP = 4), and kestose (*m*/*z* 543, DP = 3). The mass spectra obtained are consistent with the previous literature [37]. According to Zhang et al. [40], the usual degree of polymerization of garlic fructan is 58. Nevertheless, it was not possible to detect high molecular fructans with our instrument. The relative concentration of detected fructooligosaccharides was higher in aged garlic, which suggests that high molecular fructans in garlic decompose into smaller units due to the exposure of the garlic to high temperature for a long period of time. These low molecular fructooligosaccharides contribute to the characteristic sweet taste of BG. Other authors [15] also confirmed an increase of simple (reducing) sugars in BG.

Free arginine with *m*/*z* 175 [M + H]^+^ was also identified based on the production of specific fragments, with *m*/*z* 158 and 130 corresponding to a loss of NH_3_ (18) and NH_3_ + CO groups (44). However, its relative concentration increased after ageing, which implies that proteins in garlic could hydrolyze under a higher temperature during ageing, which is followed by the release of free amino acids. As reported by Kodera et al. [41], arginine is the most abundant amino acid in garlic. On the other hand, Choi et al. [6] reported that arginine is the second most abundant amino acid after tyrosine.

Amino acids, together with some sugars, can subsequently enter a Maillard reaction, leading to the production of a wide variety of substances [1]. A compound related to the Maillard reaction with molecular ion *m*/*z* 337 [M + H]^+^ producing fragments 319, 175, 158, and 130 was identified as fructosyl-arginine (Fru-Arg). This compound is formed in the early stage of the Maillard reaction and belongs to the group of Amadori compounds [20]. In addition, Fru-Arg was not detected in FG. On the other hand, it was reported that Fru-Arg is the most abundant Amadori compound in BG and has several biological properties, such as antioxidant activity, and could act as an inhibitor of the angiotensin I converting enzyme [42].

Two peaks with *m*/*z* at 231 [M + H]^+^, producing the same fragment at *m*/*z* 158, were identified as isomers (1R, 3S and 1S, 3S) of alkaloid 1-methyl-1,2,3,4-tetrahydro-β-carboline-3-carboxylic acid (MTCA). These compounds are probably formed via condensation of L-tryptophan and some aldehydes or oxo-acids in the Maillard reaction and belong to the group of β-carbolines [1]. Both isomers were detected only in BG. These compounds possess hydrogen peroxide scavenging activity, but when compared to quercetin or catechin, the scavenging activity is much lower [34].

Concerning peptides, three γ-glutamyl were detected, namely γ-glutamyl-S-allyl-l-cysteine (*m*/*z* 291 [M + H]^+^) with fragments at *m*/*z* 162 and 145; γ-glutamyl-S-(trans-1-propenyl)-l-cysteine (*m*/*z* 291 [M + H]^+^) with mass spectra at *m*/*z* 274, 162 and 145; and γ-glutamyl-phenylalanin (*m*/*z* 295 [M + H]^+^), producing the following spectra: *m*/*z* 166 and 120. These molecular ion masses and spectra match with data published previously [32,33,43,44]. The first two mentioned peptides containing sulfur serve in garlic as important storage peptides and are biosynthetic intermediates for a plethora of corresponding organosulfur compounds, including those with sensory importance, e.g., allicin [45]. These organosulfur compounds, together with γ-glutamyl-phenylalanin, have beneficial effects in human tissue, including antioxidant, anticancer, antinociceptive, antiplatelet, and anti-atherosclerotic activities [46]. Unfortunately, the relative concentration of γ-glutamyl peptides was lower in BG and γ-glutamyl-S-(trans-1-propenyl)-l-cysteine was detected only in FG.

A peak with molecular ion at *m*/*z* 163 [M + H]^+^ eluting at the end of the gradient with a dominant product ion at *m*/*z* 73 was later identified as allicin. Its relative concentration after ageing was nearly undetectable compared to FG. Despite the fact that allicin has certain positive biological properties [47], its presence in BG is not suitable in terms of sensory properties, as allicin, together with other compounds, gives garlic a typical pungent flavor [45]. Although it is known that allicin is unstable and undergoes rapid decomposition [43], Zhang et al. [3] reported detectable amounts in BG even after 69 days of ageing at 60 °C.

It is interesting that we did not detect any polyphenolic compounds, neither in the FG nor in the BG, in spite of the fact that a significant increase in total polyphenols was measured using the Folin–Ciocâlteu phenol reagent in all varieties after ageing. The results are in line with a previous study by Molina-Calle et al. [35], who also did not identify polyphenolic compounds using a high-resolution quadrupole time-of-flight mass spectrometer. According to Corzo-Martínez et al. [45], flavonoids, which are abundant, e.g., in onion, are practically absent in garlic. In addition, Beato et al. [48] detected only very low levels of phenolic acids, reaching a maximum of 2.9 mg/kg of DM for caffeic acid. The only explanation could be that some polyphenolic compounds formed during the Maillard reaction. For example, Matsutomo et al. [34] identified several dilignols in aged garlic extract possessing antioxidant activity. However, these compounds were not detected in BG. As mentioned in Section 3.2, it is described in the literature [29] that the Folin–Ciocâlteu phenol reagent could sometimes overestimate the result because the reagent may react with other interfering compounds (e.g., amino acids).

### 3.4. Color and Texture Analysis of Black and Fresh Garlic and Sensory Analysis of Black Garlic

**Figure 1:**
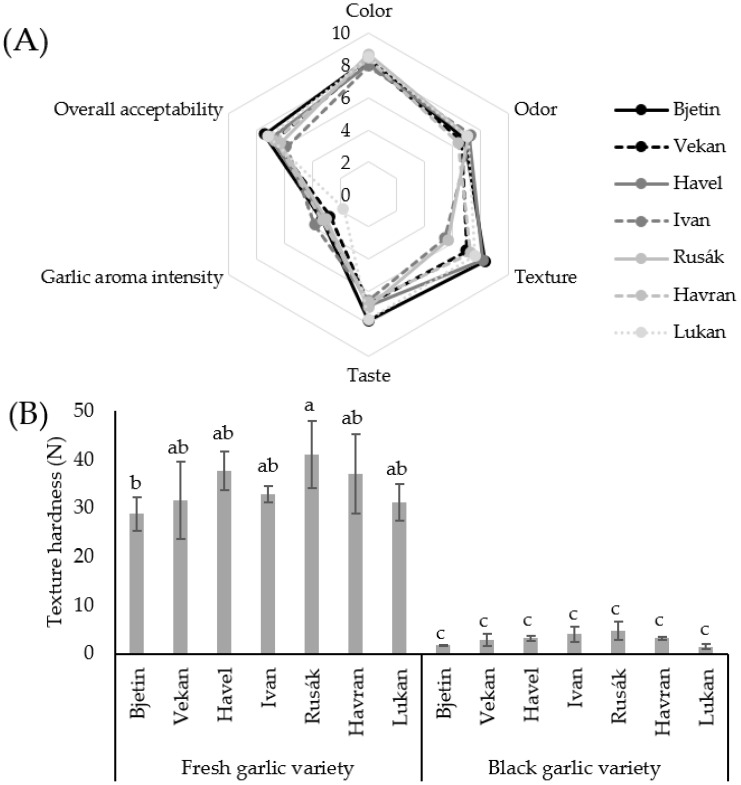
Sensory analysis (0 = dislike extremely, 10 = like extremely) of different black garlic varieties (**A**) and hardness of texture of white and black garlic varieties (**B**). ^a–c^ bars, representing means of texture hardness (*n* = 4) ± standard deviation, with different letters differ significantly (*p* < 0.05).

BG is interesting not only because of its chemical composition and biological activity, but also because it has highly remarkable organoleptic properties. It acquires a sweet-and-sour taste and a very soft, chewy, and jelly-like texture during the ageing process [49]. The pleasantness of the color, odor, texture, and taste and the intensity of the garlic aroma and the overall acceptability of the seven BG varieties were evaluated in a sensory analysis. The results of the evaluation are depicted in Figure 1A. Although the panelists perceived differences between all the varieties in all the sensory attributes, they were not statistically significant (*p* > 0.05), with the exception of texture, where the Rusák and Lukan varieties received a significantly (*p* < 0.05) lower sensory score than the remaining varieties. In terms of overall acceptability, the analysis did not reveal one variety that would be better than the others.

BG should not have as strong a garlic aroma as FG. Nevertheless, the panelists recorded that the garlic aroma was still present in all the BG samples; however, the intensity was relatively low. Although our ageing temperature was set between 70–80 °C, which is the temperature that is ideal for BG with good sensory properties, pungent garlic aroma is usually present in BG that was aged at 60 °C or for a shorter time period [3]. The reason why the garlic aroma was still perceived in our samples could be that the Czech garlic varieties have a slightly higher pungency than varieties originating from other countries [12].

It is essential to know that the variety of garlic may be an important factor affecting the sensory characteristics of BG. Recently, there have been two papers published regarding the influence of the garlic variety and storage time [14,15] and one focusing on the influence of the cultivation system (conventional vs. organic) [13] on selected quality parameters of BG. However, none of them involved a sensory assessment in the experiment. On the other hand, several papers have been published describing how the different ways of the ageing process can affect the sensory properties of BG, e.g., [3]. Sensory analysis results provide the evidence that the variety of garlic may not be the most important parameter (except for texture) for the sensory quality of BG. It is possible that processing conditions (temperature, humidity, and time) play a greater role in terms of the sensory properties of BG [5].

In addition to the sensory analysis, texture was also analyzed using a texture analyzer. The texture of BG can be characterized as soft or jelly-like and is unambiguously different from FG, whose texture is hard and stiff. The hardness of the texture of the FG and BG varieties is presented in Figure 1B. It is clearly shown that the hardness of garlic cloves was significantly affected by the ageing process as the average hardness of the FG samples was 34.29 N, but the BG samples had a much softer texture (average 3.01 N). Thus, there was approximately an 11-fold decrease in hardness.

This decrease caused by the ageing process could be related to the disruption of the cell wall polysaccharides of garlic due to a higher temperature [9]. Contrary results were presented by Karnjanapratum et al. [10], who described that the hardness of garlic cloves increased over 18 days of processing from 25.52 to 107.77 N. It is very interesting because the increase in hardness during ageing can be a sign of excessive loss of water and subsequently proof of the intensive drying of garlic cloves. However, based on our experience, values higher than 35 N reflect the firm and stiff texture of garlic cloves.

Differences between the garlic varieties (both before and after ageing) were also observed; the hardness ranged from 31.12 to 41.00 N for the FG samples and from 1.47 to 4.73 N for the FG samples. However, these differences were statistically significant (*p* < 0.05) only for the FG samples, but for the BG samples, the differences were not significant (*p* > 0.05). In other words, although there can be differences between the FG varieties, the differences are somewhat diminished after ageing. Considering the hardness and the results from the sensory analysis (Figure 1A), the pleasantness of the texture seems to be highly associated with the hardness of a BG clove (r = −0.793, *p* < 0.05). The panelists evaluated harder samples with a lower sensory score; on the other hand, softer samples were evaluated better.

It is, however, hard to compare our results with those of the other authors as there are no publications describing the influence of variety on the hardness of BG. According to Ríos-Ríos et al. [5], there is generally a lack of BG-texture parameters described in the literature. The authors, nevertheless, suggest that if the moisture of BG is between 40 and 50%, it would have a desirable soft texture, and when the moisture is below 40%, the texture is too hard. Results of this study show that hardness can also be a good predictor of sensory quality.

Color and appearance are one of the most important parameters of food quality that consumers evaluate as a first factor that subsequently affects the decision whether to eat the product or not. Color analysis of garlic samples, expressed in the CIE L*a*b* system, revealed that both factors (variety and ageing) significantly influence all color parameters (L*, a* and b*), as shown in Table 4.

During the ageing process, the color of all the samples turned from white to dark brown or black. The black color, which is a good indicator of the quality of the BG, is caused by the formation of brown color compounds usually associated with the advanced stage of the Maillard reaction [5].

Regarding lightness (L* value), values ranged between 82.67 and 87.44 in the FG samples, with an average value of 83.95 and between 10.64 and 26.51 in the BG samples (average 18.11). Thus, the lightness decreased almost five times during the ageing process, which was found to be statistically significant (*p* < 0.05). This result is in line with earlier reports [10,13].

No statistically significant differences (*p* > 0.05) were observed between the FG varieties. On the other hand, significant differences (*p* < 0.05) were observed between the BG samples.

The redness (a* value) of the garlic samples was also affected by variety and ageing (*p* < 0.05). The FG samples had lower redness (average 1.42) than the BG samples (average 4.63); thus, the value increased significantly during the ageing process. However, only minor differences (0.58–2.09) were measured between the garlic varieties before ageing (FG samples), but substantial differences were observed in the aged BG samples, where the redness ranged from 1.26 to 9.50.

As in the case of redness, yellowness (b* value) was also affected by both factors, ageing and variety, significantly (*p* < 0.05). The ageing process caused a drastic decrease of the b* value from 22.03 (FG) to 4.69 (BG) on average. Although no significant differences (*p* > 0.05) were observed between the FG varieties, after the ageing process, the BG varieties differed significantly with a very wide range (1.55 to 10.82).

### 3.5. The Relationships between Properties of Fresh and Black Garlic

Although two-way ANOVA can reveal the statistically significant influence of different factors (in this case, the influence of the ageing process and variety) on the quality parameters of BG, we wanted to find out the association between the parameters of FG and BG. The relationship between the moisture, pH, total soluble solids, DPPH, FRAP, TPC, hardness, L, and the a* and b* values of the FG and BG varieties is shown in Table 5.

The correlation analysis brought interesting results. Among all the examined traits, only moisture, hardness, and lightness (L* value) were found to be statistically significant (*p* < 0.05). In other words, these parameters of BG seem to be tightly related to the properties of FG before processing, e.g., the moister the FG is, the moister the BG is (r = 0.814, *p* < 0.05).

It was also found that there was a high positive correlation between the hardness of the FG and BG samples (r = 0.774, *p* < 0.05), thus it can be stated that the hardness of the BG is strongly affected by the hardness of the FG. It can be pointed out from the results that it could be recommended to choose an FG variety with a softer texture to produce BG with good texture properties.

An interesting fact is that a high correlation with statistical significance was found between the L* value of the fresh and BG samples (r = 0.804, *p* < 0.05). This means that the lighter the FG is, the lighter the BG is. The lightness of white garlic seems to be a relatively good predictor of the lightness of the BG.

On the other hand, the association between pH, total soluble solids, DPPH, FRAP, TPC, and the a* and b* values of FG and BG were relatively small and without any statistical significance (*p* > 0.05). Regarding the association between the DPPH values of the FG samples and the BG samples, only a small and statistically insignificant correlation (r = 0.588, *p* > 0.05) was found, which means that the antioxidant activity of BG is not related to the antioxidant activity of FG. Thus, it seems that the antioxidant activity of finished BG is affected by other parameters, and the antioxidant activity of FG is not a suitable parameter for the prediction of the antioxidant activity of BG. This statement can be also applied to the FRAP assay (r = 0.355, *p* > 0.05). A similar trend can be seen in a paper published by Toledano Medina et al. [15]. It could be hypothesized that other parameters, such as the concentration of organosulfur compounds and amino acid composition and the content of reducing sugars, could act as a predictor for the increase in antioxidant activity in BG. As mentioned by Yilmaz and Toledo [50], reducing sugars and amino acids enter the Maillard reaction, which could result in the production of compounds with high antioxidant activity.

Thus pH, total soluble solids, DPPH, FRAP, TPC, and the a* and b* values of BG are probably independent from FG, which implies that other factors play a more important role and influence these parameters more. Sunanta et al. [14] reported similar results. According to the above-mentioned authors, changes in the physicochemical traits were independent from the genotype of the garlic sample.

Additional correlation analysis, showing interesting associations, is presented in the Supplementary Material (Table S1). For instance, we found a significant correlation between the yellowness (b*) of FG and the pleasantness of odor of BG (r = 0.76, *p* < 0.05). However, we have not found any relevant explanation in the current literature.

In general, more research should be done to estimate the associations between the FG and BG parameters. Moreover, a higher number of varieties from different growing locations (countries or continents) should be examined to fully understand the relationships between all the evaluated parameters.

## 4. Conclusions

In this study, seven varieties of FG (namely Bjetin, Vekan, Havel, Ivan, Rusák, Havran, and Lukan) were examined and used to manufacture BG to evaluate the effects of different FG varieties and the ageing process on the properties of BG. Based on the results obtained, the following conclusions can be drawn. It is evident that the studied parameters were positively affected by the ageing process in all varieties, mainly the antioxidant activity, and the samples of BG obtained favorable attributes. There is a significant influence of variety on the physicochemical parameters of BG, namely on moisture, total soluble solids and total polyphenol content, pH, antioxidant activity, texture, and color. The ageing process also significantly affected all the studied traits of garlic, which is in line with previous reports [14,15].

In addition to the physicochemical parameters, sensory properties were also evaluated in all seven BG varieties, and the results show that the varieties of FG used in this study could have only a minor impact on the sensory properties of FG, except for the texture, where significant differences were found.

Moreover, in the correlation analysis, it was found that most of the studied parameters of BG seem to be independent of the properties of FG, with the exception of the moisture content, lightness, and hardness of the garlic cloves. Thus, it implies that most of the studied traits of FG cannot be used as a predictor of BG quality. For example, the FG with the highest antioxidant activity compared to other varieties does not have to have the highest antioxidant activity after ageing.

This suggests that further research is still needed to find out what parameters affect the quality of BG (e.g., amino acid composition, reducing sugars concentration, or organosulfur compounds) as the current knowledge is insufficient in this aspect. Moreover, experiments involving a higher number of varieties originating from European or Asian countries are needed for a deeper understanding of the influence of the variety of FG on the quality of BG.

## 5. Patents

The patent resulting from this work is Czech Patent No. 308 653: Method of preparing black garlic with antioxidant activity and black garlic prepared in this way.

## Figures and Tables

**Table 1:** Moisture content, pH, and total soluble solids of fresh and black garlic varieties.

Variety	Moisture Content (%)		pH		Total Soluble Solids (°Brix)	
	Fresh	Black	Fresh	Black	Fresh	Black
Bjetin	60.18 ± 0.64 ^abA^	36.95 ± 0.93 ^cdB^	6.06 ± 0.02 ^abA^	4.45 ± 0.03 ^aB^	39.67 ± 0.06 ^aB^	52.00 ± 0.00 ^bA^
Vekan	58.62 ± 1.60 ^bcA^	34.28 ± 1.26 ^dB^	6.05 ± 0.01 ^abA^	4.48 ± 0.05 ^aB^	39.00 ± 0.10 ^aB^	50.33 ± 0.58 ^bA^
Havel	57.04 ± 0.46 ^cA^	35.50 ± 1.09 ^dB^	6.11 ± 0.02 ^aA^	4.39 ± 0.02 ^abB^	37.67 ± 0.12 ^abB^	60.00 ± 0.00 ^bA^
Ivan	59.17 ± 2.18 ^bcA^	39.52 ± 1.16 ^bcB^	6.01 ± 0.03 ^abA^	4.32 ± 0.09 ^bcB^	36.67 ± 0.12 ^bB^	50.00 ± 1.00 ^bA^
Rusák	57.12 ± 1.20 ^cA^	33.52 ± 0.40 ^dB^	6.05 ± 0.01 ^abA^	4.24 ± 0.05 ^cB^	39.67± 0.06 ^aB^	61.00 ± 0.00 ^aA^
Havran	65.34 ± 2.46 ^aA^	42.52 ± 0.87 ^abB^	6.00 ± 0.00 ^abA^	4.38 ± 0.02 ^abB^	30.00± 0.00 ^cB^	51.67 ± 1.15 ^bA^
Lukan	62.25 ± 0.59 ^abA^	45.55 ± 0.91 ^aB^	5.99 ± 0.01 ^bA^	4.48 ± 0.05 ^aB^	32.00 ± 0.00 ^cB^	50.67 ± 0.58 ^bA^
Average	59.57 ± 2.44 ^B^	38.26 ± 4.48 ^A^	6.04 ± 0.04 ^A^	4.39 ± 0.61 ^B^	36.38 ± 3.68 ^B^	53.67 ± 9.53 ^A^

Results are presented as mean ± standard deviation (*n* = 3); ^a–d^ values with different superscripts within a column differ significantly (*p* < 0.05); ^A,B^ values with different superscripts within a row and the same analysis differ significantly (*p* < 0.05).

**Table 2:** Antioxidant activity (DPPH and FRAP assays) and total polyphenol content (TPC) of fresh and black garlic varieties.

Variety	DPPH (g TE/kg DM)		FRAP (g TE/kg DM)		TPC (g GAE/kg DM)	
	Fresh	Black	Fresh	Black	Fresh	Black
Bjetin	1.62 ± 0.02 ^bB^	13.12 ± 0.18 ^bA^	0.38 ± 0.01 ^B^	13.19 ± 0.29 ^cA^	3.62 ± 0.08 ^B^	16.01 ± 0.28 ^aA^
Vekan	1.32 ± 0.02 ^bB^	12.80 ± 0.53 ^bcA^	0.66 ± 0.02 ^B^	12.51 ± 0.35 ^dA^	3.82 ± 0.16 ^B^	11.60 ± 0.17 ^cA^
Havel	2.52 ± 0.09 ^aB^	14.00 ± 0.10 ^aA^	0.56 ± 0.02 ^B^	13.54 ± 0.20 ^bcA^	3.61 ± 0.15 ^B^	15.48 ± 0.25 ^aA^
Ivan	1.89 ± 0.08 ^abB^	12.28 ± 0.13 ^cA^	0.47 ± 0.01 ^B^	12.15 ± 0.17 ^dA^	3.89 ± 0.22 ^B^	14.90 ± 1.08 ^aA^
Rusák	1.52 ± 0.07 ^bB^	12.67 ± 0.18 ^bcA^	0.38 ± 0.01 ^B^	12.47 ± 0.49 ^dA^	3.76 ± 0.08 ^B^	15.59 ± 0.15 ^aA^
Havran	2.53 ± 0.10 ^aB^	14.25 ± 0.57 ^aA^	0.62 ± 0.03 ^B^	14.24 ± 0.36 ^aA^	4.61 ± 0.11 ^B^	13.42 ± 0.27 ^bA^
Lukan	1.89 ± 0.06 ^abB^	11.41 ± 0.49 ^dA^	0.48 ± 0.01 ^B^	12.16 ± 0.53 ^dA^	3.47 ± 1.01 ^B^	11.23 ± 0.22 ^cA^
Average	1.90 ± 0.52 ^B^	12.93 ± 4.33 ^A^	0.50 ± 0.12 ^B^	12.89 ± 4.79 ^A^	3.82 ± 0.37 ^B^	14.03 ± 4.44 ^A^

Results are presented as mean ± standard deviation (*n* = 4 for DPPH and FRAP; *n* = 3 for TPC); ^a–d^ values with different superscripts within a column differ significantly (*p* < 0.05); ^A,B^ values with different superscripts within a row and the same analysis differ significantly (*p* < 0.05); DM: dry matter; TE: Trolox equivalent; DPPH: 2,2-diphenyl-1-picrylhydrazyl; FRAP: ferric reducing antioxidant power; TPC: total polyphenol content; GAE: gallic acid equivalent.

**Table 3:** Tentatively identified compounds in fresh (FG) and black garlic (BG).

Compound	RT * (min)	Molecular Ion [M + H]^+^ (*m*/*z*)	Fragments (*m*/*z*)	Presence in Samples	Change after Ageing ⁑
Arginine	1.56	175	158, 130	FG, BG	↑
Fructosyl-arginine	1.57	337	319, 175, 158, 130	BG	↑
Fructooligosaccharide (Degree of polymerization = 9)	1.61	1515 [M + K]^+^	1353, 1191, 1029, 867, 705, 543, 381 [M + K]^+^	FG, BG	↑
Fructofuranosyl nystose (4× fructose, 1× glucose)	1.62	867 [M + K]^+^	705, 543, 381 [M + K]^+^	FG, BG	↑
Nystose (3× fructose, 1× glucose)	1.63	705 [M + K]^+^	543, 381 [M + K]^+^	FG, BG	↑
Kestose (2× fructose, 1× glucose)	1.63	543 [M + K]^+^	381 [M + K]^+^	FG, BG	↑
γ-l-glutamyl-S-allyl-l-cysteine	3.24	291	162, 145	FG, BG	↓
γ-l-glutamyl-S-(trans-1-propenyl)-l-cysteine	4.00	291	274, 162, 145	FG	↓
γ-l-glutamyl-phenylalanine	4.88	295	166, 120	FG, BG	↓
(1R, 3S)-1-methyl-1,2,3,4-tetrahydro-β-carbolin-3-carboxylic acid (MTCA)	6.91	231	158	BG	↑
(1S, 3S)-1-methyl-1,2,3,4-tetrahydro-β-carbolin-3-carboxylic acid (MTCA)	7.88	231	158	BG	↑
Diallyl thiosulfinate (allicin)	29.59	163	73	FG, BG	↓

* RT: retention time; ⁑: relative changes in concentrations after ageing; peak areas were compared in FG and BG on a dry matter basis; ↑: increase in concentration; ↓: decrease in concentration.

**Table 4:** Color analysis of fresh and black garlic varieties in the CIE L*a*b* system.

Variety	L* (Lightness)		a* (Redness)		b* (Yellowness)	
	Fresh	Black	Fresh	Black	Fresh	Black
Bjetin	82.67 ± 0.50 ^A^	10.64 ± 2.97 ^dB^	1.14 ± 0.16 ^B^	7.38 ± 2.86 ^abA^	22.43 ± 1.45 ^A^	7.36 ± 2.12 ^abB^
Vekan	83.13 ± 1.68 ^A^	11.35 ± 1.98 ^cdB^	1.74 ± 0.09 ^B^	9.50 ± 1.66 ^aA^	21.04 ± 0.71 ^A^	10.82 ± 2.20 ^aB^
Havel	82.75 ± 1.47 ^A^	13.35 ± 4.93 ^cdB^	1.56 ± 0.24 ^B^	3.99 ± 1.10 ^cA^	22.10 ± 0.79 ^A^	3.50 ± 1.49 ^bcB^
Ivan	83.77 ± 1.52 ^A^	23.90 ± 2.30 ^aB^	0.83 ± 0.33 ^B^	2.17 ± 1.43 ^cA^	22.27 ± 1.88 ^A^	1.66 ± 1.50 ^cB^
Rusák	82.73 ± 0.58 ^A^	17.70 ± 1.01 ^bcB^	2.02 ± 0.20 ^A^	1.26 ± 0.21 ^cB^	23.18 ± 0.94 ^A^	1.55 ± 0.46 ^cB^
Havran	85.18 ± 1.54 ^A^	23.32 ± 2.75 ^aB^	0.58 ± 0.28 ^B^	3.74 ± 1.69 ^cA^	20.92 ± 1.10 ^A^	3.17 ± 2.19 ^bcB^
Lukan	87.44 ± 0.83 ^A^	26.51 ± 1.71 ^aB^	2.09 ± 0.28 ^B^	4.40 ± 0.25 ^bcA^	22.27 ± 1.03 ^A^	4.76 ± 1.29 ^bcB^
Average	83.95 ± 1.78 ^A^	18.11 ± 6.53 ^B^	1.42 ± 0.56 ^B^	4.63 ± 3.04 ^A^	22.03 ± 0.87 ^A^	4.69 ± 7.45 ^B^

Results are presented as mean ± standard deviation (*n* = 3); ^a–d^ values with different superscripts within a column differ significantly (*p* < 0.05); ^A,B^ values with different superscripts within a row and the same analysis differ significantly (*p* < 0.05).

**Table 5:** Correlation (r) between observed parameters of fresh garlic (FG) and black garlic (BG).

Parameter	FG × BG (r)
Moisture	0.814 ^††^
pH	−0.102
Total soluble solids	0.390
DPPH assay	0.588
FRAP assay	0.355
TPC	−0.051
Hardness	0.774 ^†^
L*	0.804 ^††^
a*	0.071
b*	−0.481

The symbol ^†^ denotes statistical significance: ^†^ at *p* < 0.05 and ^††^ at *p* < 0.01; *n* = 7. DPPH: 2,2-diphenyl-1-picrylhydrazyl; FRAP: ferric reducing antioxidant power; TPC: total polyphenol content.

## Data Availability

The datasets generated for this study are available on reasonable request to the corresponding author.

## Supplementary Materials

The following are available online at https://www.mdpi.com/article/10.3390/foods10112703/s1, Table S1: correlation analysis of properties of fresh garlic and black garlic.

## References

[1] Qiu Z., Zheng Z., Zhang B., Sun-Waterhouse D., Qiao X. (2020). Formation, nutritional value, and enhancement of characteristic components in black garlic: A review for maximizing the goodness to humans. Compr. Rev. Food Sci. Food Saf..

[2] Botas J., Fernandes Â., Barros L., Alves M.J., Carvalho A.M., Ferreira I.C.F.R. (2019). A comparative study of black and white *Allium sativum* L.: Nutritional composition and bioactive properties. Molecules.

[3] Zhang X., Li N., Lu X., Liu P., Qiao X. (2016). Effects of temperature on the quality of black garlic. J. Sci. Food Agric..

[4] Ryu J.H., Kang D. (2017). Physicochemical properties, biological activity, health benefits, and general limitations of aged black garlic: A review. Molecules.

[5] Ríos-Ríos K.L., Montilla A., Olano A., Villamiel M. (2019). Physicochemical changes and sensorial properties during black garlic elaboration: A review. Trends Food Sci. Tech..

[6] Choi I.S., Cha H.S., Lee Y.S. (2014). Physicochemical and antioxidant properties of black garlic. Molecules.

[7] Moreno-Ortega A., Pereira-Caro G., Ordóñez J.L., Moreno-Rojas R., Ortíz-Somovilla V., Moreno-Rojas J.M. (2020). Bioaccessibility of bioactive compounds of ‘fresh garlic’ and ‘black garlic’ through in vitro gastrointestinal digestion. Foods.

[8] Toledano Medina M.A., Merinas-Amo T., Fernández-Bedmar Z., Font R., del Río-Celestino M., Pérez-Aparicio J., Moreno-Ortega A., Alonso-Moraga A., Moreno-Rojas R. (2019). Physicochemical characterization and biological activities of black and white garlic: In vivo and in vitro assays. Foods.

[9] Li N.Y., Lu X.M., Pei H.B., Qiao X.G. (2015). Effect of freezing pretreatment on the processing time and quality of black garlic. J. Food Process Eng..

[10] Karnjanapratum S., Supapvanich S., Kaewthong P., Takeungwongtrakul S. (2021). Impact of steaming pretreatment process on characteristics and antioxidant activities of black garlic (*Allium sativum* L.). J. Food Sci. Technol..

[11] Martins N., Petropoulos S., Ferreira I.C.F.R. (2016). Chemical composition and bioactive compounds of garlic (*Allium sativum* L.) as affected by pre- and post-harvest conditions: A review. Food Chem..

[12] Grégrová A., Čížková H., Bulantová I., Rajchl A., Voldřich M. (2013). Characteristics of garlic of the Czech origin. Czech J. Food Sci..

[13] Najman K., Sadowska A., Hallmann E. (2021). Evaluation of bioactive and physicochemical properties of white and black garlic (*Allium sativum* L.) from conventional and organic cultivation. Appl. Sci. Basel.

[14] Sunanta P., Chung H.H., Kunasakdakul K., Ruksiriwanich W., Jantrawut P., Hongsibsong S., Sommano S.R. (2020). Genomic relationship and physiochemical properties among raw materials used for Thai black garlic processing. Food Sci. Nutr..

[15] Toledano Medina M.A., Pérez-Aparicio J., Moreno-Ortega A., Moreno-Rojas R. (2019). Influence of variety and storage time of fresh garlic on the physicochemical and antioxidant properties of black garlic. Foods.

[16] Vácha F., Bedrníček J., Smetana P., Samková E., Kadlec J., Jirotková D., Tůma K. (2020). Method of Preparing Black Garlic with Antioxidant Activity and Black Garlic Prepared in This Way. https://isdv.upv.cz/webapp/!resdb.pta.frm.

[17] Brand-Williams W., Cuvelier M.E., Berset C. (1995). Use of a free-radical method to evaluate antioxidant activity. Food Sci. Technol.-Lebensm.-Wiss. Technol..

[18] Dudonné S., Vitrac X., Coutière P., Woillez M., Mérillon J.M. (2009). Comparative study of antioxidant properties and total phenolic content of 30 plant extracts of industrial interest using DPPH, ABTS, FRAP, SOD, and ORAC assays. J. Agric. Food Chem..

[19] Lachman J., Hosnedl V., Pivec V., Vaculová K., Ehrenbergerová J. (1998). Polyphenols in cereals and their positive and negative role in human and animal nutrition. Cereals for Human Health and Preventive Nutrition.

[20] Zhang X., Shi Y., Wang L., Li X., Zhang S., Wang X., Jin M., Hsiao C.D., Lin H., Han L. (2019). Metabolomics for biomarker discovery in fermented black garlic and potential bioprotective responses against cardiovascular diseases. J. Agric. Food Chem..

[21] Ahn S.J., Lee A., Min S.S., In S., Kim E., Kim H.J. (2019). Comparison of physicochemical characteristics of garlic produced from South Korea and China. J. Food Sci..

[22] Shin J.-H., Lee S.-J., Jung W.-J., Kang M.-J., Sung N.-J. (2011). Physicochemical characteristics of garlic (*Allium sativum* L.) on collected from the different regions. J. Agric. Life Sci..

[23] Xiong F., Dai C.H., Hou F.R., Zhu P.P., He R.H., Ma H.L. (2018). Study on the ageing method and antioxidant activity of black garlic residues. Czech. J. Food Sci..

[24] Liang T., Wei F., Lu Y., Kodani Y., Nakada M., Miyakawa T., Tanokura M. (2015). Comprehensive NMR analysis of compositional changes of black garlic during thermal processing. J. Agric. Food Chem..

[25] Toledano-Medina M.A., Pérez-Aparicio J., Moreno-Rojas R., Merinas-Amo T. (2016). Evolution of some physicochemical and antioxidant properties of black garlic whole bulbs and peeled cloves. Food Chem..

[26] Kimura S., Tung Y.C., Pan M.H., Su N.W., Lai Y.J., Cheng K.C. (2017). Black garlic: A critical review of its production, bioactivity, and application. J. Food Drug Anal..

[27] Moreno F.J., Corzo-Martínez M., del Castillo M.D., Villamiel M. (2006). Changes in antioxidant activity of dehydrated onion and garlic during storage. Food Res. Int..

[28] Piluzza G., Bullitta S. (2011). Correlations between phenolic content and antioxidant properties in twenty-four plant species of traditional ethnoveterinary use in the Mediterranean area. Pharm. Biol..

[29] Everette J.D., Bryant Q.M., Green A.M., Abbey Y.A., Wangila G.W., Walker R.B. (2010). Thorough study of reactivity of various compound classes toward the Folin-Ciocalteu reagent. J. Agric. Food Chem..

[30] Huang D.J., Ou B.X., Prior R.L. (2005). The chemistry behind antioxidant capacity assays. J. Agric. Food Chem..

[31] Kim J.S., Kang O.J., Gweon O.C. (2013). Comparison of phenolic acids and flavonoids in black garlic at different thermal processing steps. J. Funct. Foods.

[32] Chang W.C., Chen Y.T., Chen H.J., Hsieh C.W., Liao P.C. (2020). Comparative UHPLC-Q-Orbitrap HRMS-based metabolomics unveils biochemical changes of black garlic during aging process. J. Agric. Food Chem..

[33] Chi M.-C., Lo H.-F., Lin M.-G., Chen Y.-Y., Lin L.-L., Wang T.-F. (2017). Application of *Bacillus licheniformis* γ-glutamyltranspeptidase to the biocatalytic synthesis of γ-glutamyl-phenylalanine. Biocatal. Agric. Biotechnol..

[34] Matsutomo T., Stark T.D., Hofmann T. (2013). In vitro activity-guided identification of antioxidants in aged garlic extract. J. Agric. Food Chem..

[35] Molina-Calle M., de Medina V.S., Priego-Capote F., de Castro M.D.L. (2017). Establishing compositional differences between fresh and black garlic by a metabolomics approach based on LC-QTOF MS/MS analysis. J. Food Compos. Anal..

[36] Wang X., Yang Y., Liu R., Zhou Z., Zhang M. (2016). Identification of antioxidants in aged garlic extract by gas chromatography-mass spectrometry and liquid chromatography-mass spectrometry. Int. J. Food Prop..

[37] Zhang H., Zhu L., Chen H.W. (2014). Direct molecular analysis of garlic using internal extractive electrospray ionization mass spectrometry. Chin. J. Anal. Chem..

[38] Metlin. http://metlin.scripps.edu.

[39] MassBank. http://massbank.eu.

[40] Zhang N., Huang X., Zeng Y., Wu X., Peng X. (2013). Study on prebiotic effectiveness of neutral garlic fructan in vitro. Food Sci. Human Wellness.

[41] Kodera Y., Kurita M., Nakamoto M., Matsutomo T. (2020). Chemistry of aged garlic: Diversity of constituents in aged garlic extract and their production mechanisms via the combination of chemical and enzymatic reactions. Exp. Ther. Med..

[42] Yu J.H., Shan Y., Li S., Zhang L.F. (2020). Potential contribution of Amadori compounds to antioxidant and angiotensin I converting enzyme inhibitory activities of raw and black garlic. LWT-Food Sci. Technol..

[43] Ho S.C., Su M.S. (2014). Evaluating the anti-neuroinflammatory capacity of raw and steamed garlic as well as five organosulfur compounds. Molecules.

[44] Kim S., Park S.L., Lee S., Lee S.Y., Ko S., Yoo M. (2016). UPLC/ESI-MS/MS analysis of compositional changes for organosulfur compounds in garlic (*Allium sativum* L.) during fermentation. Food Chem..

[45] Corzo-Martínez M., Corzo N., Villamiel M. (2007). Biological properties of onions and garlic. Trends Food Sci. Tech..

[46] Yang J., Bai W.D., Zeng X.F., Cui C. (2019). Gamma glutamyl peptides: The food source, enzymatic synthesis, kokumi-active and the potential functional properties—A review. Trends Food Sci. Tech..

[47] Chung L.Y. (2006). The antioxidant properties of garlic compounds: Allyl cysteine, alliin, allicin, and allyl disulfide. J. Med. Food.

[48] Beato V.M., Orgaz F., Mansilla F., Montaño A. (2011). Changes in phenolic compounds in garlic (*Allium sativum* L.) owing to the cultivar and location of growth. Plant. Food Hum. Nutr..

[49] Martínez-Casas L., Lage-Yusty M., López-Hernández J. (2017). Changes in the aromatic profile, sugars, and bioactive compounds when purple garlic is transformed into black garlic. J. Agric. Food Chem..

[50] Yilmaz Y., Toledo R. (2005). Antioxidant activity of water-soluble Maillard reaction products. Food Chem..

