# Supplementary material for: The Use of a Thermal Process to Produce Black Garlic: Differences in the Physicochemical and Sensory Characteristics Using Seven Varieties of Fresh Garlic
Source: Foods. 2021 Nov 5;10(11):2703. doi: 10.3390/foods10112703 (PMC8623035; doi:10.3390/foods10112703)
Supplement: Supplementary file 1 [file foods-10-02703-s001.zip › foods-1419764-supplementary.pdf]

foods

Article

Supplementary material: Variety and ageing process as factors influencing selected physicochemical and sensory characteristics of black garlic

Jan Bedrníček[1]*, Ivana Laknerová[2], František Lorenc[1], Priscila Probio de Moraes[4], Markéta Jarošová[3], Eva Samková[1], Jan Tříska[5], Naděžda Vrchotová[5], Jaromír Kadlec[1], and Smetana Pavel[1]

[1] University of South Bohemia in České Budějovice, Faculty of Agriculture, Department of Food Biotechnologies and Agricultural Products' Quality, Studentská 1668, 370 05 České Budějovice, Czech Republic; bedrnj00@zf.jcu.cz (J.B.); kadlec@zf.jcu.cz (J.K.); samkova@zf.jcu.cz (E.S.); smetana@zf.jcu.cz (P.S.); lorenf00@zf.jcu.cz (F.L.).
[2] Food Research Institute Prague, Radiová 1285/7, 102 00 Praha 10 – Hostivař, Czech Republic; ivana.laknerova@vupp.cz (I.L.)
[3] University of South Bohemia in České Budějovice, Faculty of Agriculture, Department of Plant Production, Na Sádkách 1780, 370 05 České Budějovice, Czech Republic; jarosm08@zf.jcu.cz (M.J)
[4] University of Campinas, School of Food Engeneering, Department of Food Science, Monteiro Lobato n°80, 13083-862 Campinas, São Paulo, Brazil; priscilaprobio-moraes@gmail.com (P.P.M.)
[5] Global Change Research Institute CAS, 603 00 Brno, Czech Republic; triska.j@czechglobe.cz (J.T.); vrchotova.n@czechglobe.cz (N.V.)
* Correspondence: bedrnj00@zf.jcu.cz; Tel.: + 420 721 657 114

Table S1. Correlation analysis of properties of fresh garlic and black garlic.

	Parameter	Properties of Black garlic															
		Basic analyses			Antioxidant properties			Physical properties				Sensory analysis					
		Moisture	pH	TSS	DPPH	FRAP	TPC	Hardness	L*	a*	b*	Color	Odor	Texture	Taste	Garlic aroma intensity	Overall acceptability
Properties of fresh garlic	**Moisture**	0,81	0,33	-0,59	0,11	0,43	-0,44	-0,43	0,56	0,04	-0,01	0,26	-0,62	0,23	0,11	-0,18	0,22
	**pH**	-0,81	-0,10	0,69	0,47	0,22	0,56	0,19	-0,82	0,17	0,15	-0,22	0,21	0,31	-0,06	0,26	0,20
	**TSS**	-0,89	-0,20	0,39	-0,05	-0,34	0,51	0,27	-0,78	0,24	0,28	-0,11	0,34	-0,13	0,06	0,30	-0,04
	**DPPH**	0,42	-0,07	0,18	0,59	0,69	0,14	0,01	0,35	-0,40	-0,51	-0,25	-0,08	0,31	-0,26	0,17	0,04
	**FRAP**	0,08	0,42	-0,28	0,39	0,35	-0,59	-0,06	-0,03	0,41	0,39	-0,40	-0,55	0,18	-0,56	-0,10	-0,02
	**TPC**	0,20	-0,23	-0,21	0,58	0,62	-0,05	0,33	0,28	-0,16	-0,21	0,02	-0,68	-0,22	-0,62	0,43	-0,35
	**Hardness**	-0,31	-0,76	0,80	0,41	0,28	0,33	0,77	0,11	-0,68	-0,64	0,23	0,08	-0,37	-0,51	0,19	-0,45
	**L***	0,93	0,37	-0,47	-0,45	-0,13	-0,71	-0,47	0,80	-0,13	-0,12	0,22	-0,07	0,10	0,28	-0,66	0,16
	**a***	-0,23	0,10	0,39	-0,54	-0,56	-0,30	-0,09	-0,14	0,07	0,19	0,39	0,37	0,03	0,40	-0,74	0,22
	**b***	-0,23	-0,53	0,54	-0,43	-0,47	0,56	0,24	0,02	-0,53	-0,48	0,31	0,76	-0,26	0,41	0,00	-0,10

Values in the table represent correlation coefficients (r). Correlation coefficients highlighted in red color are statistically significant ($P < 0.05$). TSS: DPPH: 2,2-diphenyl-1-picrylhydrazyl; FRAP: ferric reducing antioxidant power; TPC: total polyphenol content; TSS: total soluble solids.
